# Supplementary material for: MetaRibo-Seq measures translation in microbiomes
Source: Nat Commun. 2020 Jun 29;11:3268. doi: 10.1038/s41467-020-17081-z (PMC7324362; doi:10.1038/s41467-020-17081-z)
Supplement: Supplementary file 10 — Supplementary Data 7 [file 41467_2020_17081_MOESM10_ESM.zip › File2/Confidence_High_Taxonomy/114476_out.krona.html]

Javascript must be enabled to view this page.

members
magnitude
magnitudeUnassigned
count
unassigned
taxon
rank

114476\_out

3

2
superkingdom
3

1239
phylum
3

class
3
186801

186802
order
3

541000
1
family

1263
1
genus

SRS019327\_contig\_number\_36288

2
family
186803

43996
1
genus

43997
1

SRS018591\_contig\_number\_2572
species

1
species

SRS051791\_contig\_number\_16361
712991
